# Flexible, Self-Determined… and Unhealthy? An Empirical Study on Somatic Health Among Crowdworkers

**DOI:** 10.3389/fpsyg.2021.724966

**Published:** 2021-12-02

**Authors:** Katharina D. Schlicher, Julian Schulte, Mareike Reimann, Günter W. Maier

**Affiliations:** ^1^Department of Psychology, Work and Organizational Psychology, Bielefeld University, Bielefeld, Germany; ^2^Research Institute for Cognition and Robotics (CoR-Lab), Bielefeld University, Bielefeld, Germany; ^3^Department of Sociology, Social Structure and Social Inequality, Bielefeld University, Bielefeld, Germany

**Keywords:** crowdwork, crowdsourcing, gig work, digital work, somatic health, regeneration, leisure, work-life balance

## Abstract

Crowdwork is a new form of digitally enabled work in which organizations assign tasks to an anonymous group of workers *via* platform intermediaries. For crowdworkers, crowdwork offers both opportunities and risks. On the one side, crowdworkers enjoy high flexibility on when, where, and how much to work. On the other side, risks comparable to other forms of atypical employment arise: no labor regulation, unstable income, and uncertainty about whether enough tasks are available. Regulation of working hours lies within the crowdworkers’ own authority. Also, crowdwork in industrialized nations is often conducted during leisure times as a side-job to some other kind of employment. In accordance with Conservation of Resources Theory, we state that when leisure time gets used up with crowdwork, regeneration cannot occur and health declines. On a sample of *N*=748 German crowdworkers recruited from four different platform types, we analyzed whether participation in crowdwork is linked to increased somatic symptoms compared to regularly employed personnel. We found that crowdworkers show significantly increased somatic symptoms as compared to a German norm sample, that are stable across different kinds of tasks and platforms, gender, and age groups, and that is statistically due to the extent of participation in crowdwork. Specifically, we found that total work hours per week were not associated with an increase in somatic symptoms, but we did find associations with strain-based work–family conflict and the primary motivation to do crowdwork being to earn money. Consequences for research and labor regulations are discussed.

## Introduction

Since the emergence of an internet-based, digital job market, crowdwork (CW) has increasingly become a relevant new form of work (Berg et al., 2018; Watson et al., 2021). CW describes a form of online mediated, paid work where contracting requesters (the crowdsourcers) post single and briefly contracted work tasks online to an anonymous group of interested workers (the crowd or crowdworkers) *via* an open call on an intermediary (the platform; Schulte et al., 2020). A hallmark of CW is that the work is little regulated concerning who can participate on the platform as crowdsourcers or crowdworkers or concerning the duration of task commitments, resulting in a fully flexible job market. It is estimated that 6–12% of the European workforce work or intend to work in CW (Pesole et al., 2018; Serfling, 2019).

Due to the novelty of the concept, little is known about working conditions and the consequences of conducting CW from the perspective of the crowdworkers, especially consequences on crowdworkers’ health (Pesole et al., 2018). It could be assumed that CW is part of atypical work arrangements because the work is not regulated by law, the crowdworkers are barely organized, and their status (self-employed vs. employee of a platform) remains unclear (Felstiner, 2011; Berg et al., 2018; Pesole et al., 2018). Moreover, CW is low on labor protection, and social security is the crowdworkers’ own responsibility (de Stefano, 2016). Extensive literature on atypical work arrangements has already proven their negative impact on the individual’s perceived stress, mental health, musculoskeletal problems, and other physical health problems (e.g., Quinlan et al., 2001; Sverke et al., 2002; Tavares, 2017). Finally, qualitative research on crowdworkers’ perception of working conditions indicates increased stress levels due to overwork and inhibited regeneration from work (Huws et al., 2017).

The present empirical study aims to answer the research question whether crowdworkers have a higher risk of impaired health due to their participation in CW and why this relation occurs. To answer the research questions, we first draw parallels to related forms of atypical work arrangements by reviewing the literature on the health effects of telework, short-term contracted work, and self-employment. Telework in particular is of interest, as the Covid-19 pandemic has shown the importance of digital and flexible forms of work, such as CW, that can be done from home (Belzunegui-Eraso and Erro-Garcés, 2020; Spurk and Straub, 2020). Second, we derive theoretical assumptions on the mechanisms of the relation of CW and health by turning to findings of qualitative research and principles of conservation of resources theory (COR; Hobfoll et al., 1990, 2018), one of the most prominent stress theories. We then test our assumptions in an empirical analysis of *N*=748 German crowdworkers from four different platform types. We contribute to the literature in the following way: First, we sketch how the occurrence of somatic health symptoms is distributed among crowdworkers from different platforms to cover the whole bandwidth of tasks of an industrialized nation in CW. Second, we show that impaired somatic health of crowdworkers is not just due to a selectivity of the employment group that are rather attracted by the flexibility of the crowdwork market, but that impaired somatic health is directly associated with their engagement in CW. Third, we provide a theoretically derived model on how impairment of crowdworkers’ somatic health occurs. And fourth, we derive health-promoting design options for CW.

## Health Impairment Effects of CW

CW distinguishes from other forms of employment by being contracted and limited in compensation and duration of completion to single tasks (Schulte et al., 2020; Watson et al., 2021). The term CW must be differentiated from other forms of online work: the gig economy and crowdsourcing. Whereas the gig economy encompasses paid tasks that are mediated *via* an online platform, but are accomplished remotely (Schulte et al., 2020; Watson et al., 2021), crowdsourcing describes a form of participatory online activity that can have other forms of compensation than payment, for example goods or personal skill enhancement (Estellés-Arolas and González-Ladrón-de-Guevara, 2012). CW is limited to paid online work only (for a detailed overview, see Schulte et al., 2020). Tasks on CW platforms can vary in their content, level of duration of completion, qualification requirements, and compensation (Leimeister et al., 2016; Howcroft and Bergvall-Kåreborn, 2019).

Also, demographic characteristics of crowdworkers are heterogeneous. Crowdworkers of industrialized nations rather participate in CW as a side-job to their regular contracted work (up to 47% of all crowdworkers; Serfling, 2019). This can be explained by the primary motivation to generate income by CW. Payments for CW tasks suffice as a sole income for workers from developing and emerging countries (Ipeirotis and Stern, 2010). Another group of crowdworkers conduct CW as a side-job to some education program or in retirement (Serfling, 2019). Furthermore, there are indications that the high flexibility of the CW market offers suitable working conditions for health impaired workers, who cannot or not fully take up regular work (Zyskowski et al., 2015; Hara and Bigham, 2017). Overall, the CW population consists of rather young, male, well-educated, family-bound cohorts (Pesole et al., 2018; Serfling, 2019).

### Health Effects of CW and Related Forms of Work

Consequences of CW on crowdworkers’ health have rarely been investigated, even though CW is receiving increasing attention in research (for an overview: e.g., Ghezzi et al., 2018) and governmental reports (e.g., Pesole et al., 2018). Knowledge on physical health and psychological well-being are critical to determine regulatory requirements and design options for CW. We start by reviewing the scarce literature on CW health and well-being and continue to draw parallels to atypical work arrangements that might help to determine whether and which health risks might arise from participation in CW.

The health of crowdworkers has been addressed in research mainly to determine the demographics and status quo of crowdworkers. Shapiro et al. (2013) concluded that the prevalence of depression and general anxiety disorders were comparable to a general population, but that social anxiety was higher in CW populations, though social anxiety is generally higher in internet-user populations. Qualitative research also reveals the health hazards of CW imposed by long working hours at the computer, for example neck and back pain, risks of accidents in mobile sourcing tasks, depression, and work–family conflict resulting from stress at work (Huws et al., 2017). CW is perceived as stressful due to the randomness of the kind of next task to be done, the constant unpaid search for new tasks, the need to respond quickly to newly arriving tasks, the unpredictability of net income due to the unstable amount of available work, fear of negative customer ratings that might affect the chances of receiving additional tasks (Huws et al., 2017), and the lack of guarantee of actually being paid for a completed task because crowdsourcers can still reject payment (Ho et al., 2015).

Parallels to other atypical work arrangements shed further light on the possible related health effects of CW. Atypical employment in general is associated with increased insecurity of continuing work, less control over work processes such as pace and organization of work and assignment of tasks and methods, lower income and less benefits, lower work-role status as perceived by peers and the organization, less social support at work, exposure to physical health hazards, and less training and career advancement opportunities (Tompa et al., 2007). Atypical employment is associated with lower levels of both objective and subjective health (Quinlan et al., 2001), especially when uncertainty of the employment relation and efforts for future employment are high (Lewchuk et al., 2008). Specific comparisons can be drawn to the atypical work arrangements of telework, temporary work, and self-employment.

With telework, CW shares the commonality that work is performed from a technological medium such as the computer without social contacts in person. Like CW, telework has been associated with certain risk factors of lowered health, such as lowered boundaries between home and work, a tendency to overwork, and social isolation (Tavares, 2017). Furthermore, there is potential for conflicts in the form of lack of support, resentments between colleagues, and lower career progression. Also, telework settings often offer inadequate work equipment (Tavares, 2017). The effects increase the more hours an employee works in telework settings (Gajendran and Harrison, 2007). Although telework arrangements also have positive health effects, due to the association with higher levels of autonomy and control over one’s work life (Gajendran and Harrison, 2007; Tavares, 2017), certain negative outcomes have been reported: stress, depression, and musculoskeletal problems (Tavares, 2017). Moreover, recent research (López-Igual and Rodríguez-Modroño, 2020) has observed a shift in teleworkers’ profiles toward temporary, lower-paying jobs, which increases their similarity to CW.

With temporary employment, CW shares the short durations of contracts. Whereas temporary employment involves contracts of at least several months in duration, CW contracted tasks can be completed in mere seconds. Therefore, both forms of work lead to insecurity in planning financial and personal investments. In a meta-analysis, this insecurity proved to have small effects on physical health, but medium sized effects on mental health (Sverke et al., 2002). Glavin (2015) found that while temporary perceptions of job insecurity were not related to self-rated poor health, persistent experiences of insecurity led to greater distress. The effect was similar for men and women, but more relevant for older age groups. Virtanen et al. (2005) found higher overall psychological morbidity and musculoskeletal disorders compared to regular contracted workers. The effect was stronger with increased instability of the temporary work relation and the lower the unemployment rates and numbers of temporary workers in the specific country were.

Many crowdworkers either conduct CW as a side-job to another entrepreneurship or are self-employed *via* their CW activities. The majority of platforms classify crowdworkers as independent contractors with self-employed status (Berg, 2015) and this is how crowdworkers often see themselves (Pesole et al., 2018). CW is comparable to self-employment regarding low labor protection, employment insecurity, and individual responsibility for social security (de Stefano, 2016). Even though the self-employed often appear healthier than comparable employed groups, this relation might occur because healthier workers rather choose to be self-employed (Rietveld et al., 2015). Indeed, self-employment is associated with increased stress and decreased physical health, because self-employed individuals often place high demands on themselves in order to achieve economic success (Cardon and Patel, 2015). In addition, Binder and Coad (2016) found that only voluntary self-employment is associated with increased work, life and health satisfaction, whereas involuntary self-employment did not show this relation. Crowdworkers, of whom many will have taken up the activity because other regular-employed job opportunities were not available or less attractive in their respective life circumstances, likely need to be counted to the involuntary self-employment group. Still, both groups have in common that self-employment leads to a decrease in leisure-time satisfaction (Binder and Coad, 2016).

In conclusion, the comparison with related forms of atypical employment makes it reasonable to assume that participation in CW leads to lowered health, and is in particular associated with musculoskeletal problems and pain, depression, and anxiety, which in turn are associated with somatic symptoms (Gierk et al., 2014). Therefore, we hypothesize that doing CW is associated with increased somatic symptoms compared to regular employment forms.

*H1*: Doing CW is associated with increased somatic symptoms.

### Explaining the Health Risk of CW With Conservation of Resources Theory

*“Well I do not think I’m as happy as I used to be, because I have no free time. Anytime I am free, I actually have to work for [name of platform]. It does not feel nice. It just feels more, I would say I’m more depressed. But what can you do? That’s the way it goes. More stressed, I would say.” (Serkan, 48, UK;* Huws et al., *2017**, p. 47)*.

In qualitative research, from which the above statement is retrieved, CW is described as demanding because it limits time for regeneration and leisure, and in effect leads to family conflict and depression (Huws et al., 2017). This finding is in line with research showing that most European crowdworkers perform CW as a side-job to their regular employment (Serfling, 2019), often in overtime and above allowed maximum hours in undeclared labor (Baumann and Klotz, 2017). Therefore, crowdworkers use their free time on work-related tasks that would otherwise be used for regeneration from work.

The effect of increased stress in crowdworkers due to lowered regeneration times can be explained with COR (Hobfoll et al., 1990, 2018). COR states that every person possesses several personal, social, or material resources, for example a sense of self-efficacy, support from partners and friends, or financial goods. In demanding life phases, these resources get used up. COR then explains the occurrence of perceived stress by the loss or threat of loss, or a failure to gain key resources (Hobfoll et al., 2018). In the case of CW, resource loss is equivalent to the limited regeneration time. Other threats of resource loss are job insecurity, limited control over work processes, lower income, little social support at work, and limited career advancement opportunities (Tompa et al., 2007). Resource loss is thereby of higher impact than resource gain, as initial resource loss is likely to lead to a further downward spiral of resource losses (just as resource gain would lead to an upward spiral). Because resources need to be invested to gain further resources (Halbesleben et al., 2014), for example investing time for meeting friends to earn their emotional support, a resource loss of free time due to engagement in CW activities during leisure time inhibits this option. The effect varies individually as individuals with greater resources are less vulnerable to resource loss (Hobfoll et al., 2018). Therefore, the crowdworkers at high health risks are those with limited resources to begin with and additional resource threat due to limited regeneration time.

References to the relation between crowdworkers’ regeneration and health can be drawn from research. Dahlgren et al. (2006) found lower ratings of sleep quality, recuperation, and more fatigue the next day when study participants had to perform overtime work, and Tucker et al. (2008) found less satisfaction and rest in an experimental setting in which additional work-related tasks were performed in the evening, compared to quiet or active leisure activities. Dahlgren et al. (2005) tested the effects of intra-individual differences of self-rated high- and low-stress work weeks. They found that high-stress work weeks were characterized by longer working hours, reduced total sleep time, and perceived stress. Cortisol levels of participants showed a flattened pattern as compared to the low-stress week, with higher levels during the evening (interpreted as increased arousal leading to sleeplessness) and lower levels in the morning (interpreted as exhaustion due to sleeplessness the night before). Long-term increases of cortisol levels are associated with suppressed testosterone and other immune system activity, therefore demonstrating a vulnerability for the onset of diseases (Dahlgren et al., 2005). When crowdworkers see their regeneration time limited over a longer period because they work in CW tasks during their free time, then according to these results they should experience health-impairment effects.

The relation of health and regeneration is explained by different processes, namely (a) psychological detachment from work, (b) relaxation through times of positive affect, low-activity leisure activities such as reading, (c) mastery experience through high-activity leisure activities such as learning a new hobby, and (d) control during leisure time to choose the preferred option (Sonnentag and Fritz, 2007). Because the varying kinds of leisure such as active vs. passive activities showed no significant difference, Tucker et al. (2008) concluded that the mental demands of the actual job plus evening work-related tasks accumulate and in effect increase stress levels. Multilevel within-person analysis showed that extensive work-related activities during recovery periods led to decreased levels of well-being before going to sleep as compared to low-effort, social, or physical leisure activities. Furthermore, there were no interaction effects found, as a combination of work-related activities and leisure activities in the same evening or compensation of work-related tasks on one evening by leisure activities the next did not eliminate the negative effect (Sonnentag, 2001). Therefore, there does not seem to be a singular pattern of leisure activities that helps employees recover from work, rather psychological states such as detachment and relaxation need to be achieved in order to distance from stress (Sonnentag and Fritz, 2007). For most crowdworkers, the main reason to participate in CW is to earn money and CW is therefore rather perceived as work than as detachment. Only few crowdworkers indicate their motivation is to pass the time (Kaufmann et al., 2011). Also, crowdworkers must work when tasks are available and therefore find it harder to choose when to regenerate, and the constant contest with other crowdworkers for the best tasks can lead to a tendency to accept too many tasks, promise tight deadlines, and work late (Wood et al., 2019). When crowdworkers perform work-related tasks beside their main job and therefore fill their leisure time with additional work activities, they might not be able to find sufficient time to reach the psychological state of recovery from work stress.

As an indicator of a distorted leisure and regeneration behavior following long work hours we refer to increased work-life conflict (Abendroth et al., 2014), as inhibited regeneration time in crowdworkers could express itself in time-conflicts with partner, friends, and leisure activities, resulting in mental irritation for the crowdworkers. Work-life conflict consists of two facets: time-based work-life conflict and strain-based work-life conflict. Time-based work-life conflict means that due to a heavy workload, the affected individuals cannot find the time resources to engage with their family and other private activities. When crowdworkers work overtime or cannot choose when to work except for times reserved for private activities (Wood et al., 2019), they may perceive time-based work-life conflict. Strain-based work-life conflict refers to not having energy during leisure time for activities that would be necessary for recovery because the energy has already been used for work. Therefore, work-life conflict is a specific indicator of distorted regeneration related to crowdwork characteristics.

Crowdworkers in industrialized nations are more likely to practice CW as a side job to another occupation (Serfling, 2019), so it is not just the number of hours spent doing CW that leads to deterioration in somatic health, but rather the combination of hours worked in crowdwork and hours worked in the other occupation overall that leads to the adverse health effects. The singular effects of either could prove unproblematic, but the combination of the two, mostly performed in undeclared work, could lead to the adverse health effects. Therefore, we test for the overall effect of total work hours, not just the hours spent on CW.

*H2*: A high number of total work hours per week is positively associated with increased somatic symptoms in crowdworkers.

*H3*: The relation of total work hours per week and somatic symptoms is mediated by time-based and strain-based work-life conflict.

A measure that describes the role of crowdwork in the detrimental effect of total work hours on health is the share of crowdwork that can have two contrasting effects. A higher share of crowdwork in total work hours as compared to some other form of employment can be resource-depleting, because the more hours crowdworkers participate in CW, the more they are confronted with its drawbacks: working hours, working times, and place are not regulated, high competition on the platform, and long search times for suitable tasks urges crowdworkers to work at less favorable times and take up a high amount of work (Wood et al., 2019). A higher share of CW can also be resource-enhancing: the chance to design CW that is flexible to an individual’s schedule allows more work-life-balance (e.g., Huws et al., 2017). With a view to the health effects of other forms of atypical employments, we agree with Wood et al. (2019) that a higher share of CW in total work hours per week is related to increased somatic symptoms. Because we expect CW to be associated with work-life conflict due to limitations on the ability to choose when and where to work (Wood et al., 2019), higher shares of CW in total work hours will increase conflict.

*H4*: The relationship of total work hours and the mediators a) time-based work-life conflict, and b) strain-based work-life conflict, and the c) dependent variable somatic health will be moderated by share of CW in total work hours.

Finally, the primary motivation to earn money through CW explains the detrimental effects of long work hours on health (e.g., Brawley, 2017). When many work hours including CW have to be completed in order to achieve a reasonable income, a crowdworker will work beyond healthy limits and neglect times reserved for meetings with friends and family. Therefore, we hypothesize that a stronger motivation to earn money with CW is associated with poorer health, which is also due to a conflict between work and family.

*H5*: The relationship of total work hours and the mediators a) time-based work-life conflict, and b) strain-based work-life conflict, and the c) dependent variable somatic health will be moderated by motivation.

**Figure 1:**
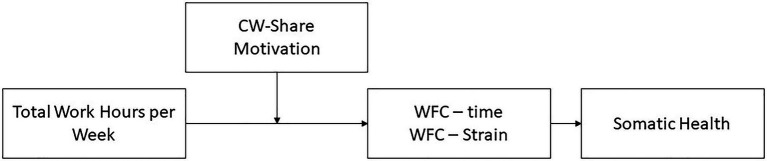
Model of Crowdworker General Somatic Health. WLC-time/−strain=time-based and strain-based work-life conflict, CW-Share=Share of crowdwork in total work hours; Motivation=primary motivation to earn money through CW.

Model assumptions are summarized in Figure 1.

## Materials and Methods

### Sample

We recruited participants from four exemplary CW platforms to gain insight into the full scope of CW in Germany. For platform selection, a systematic analysis of all German CW platforms was conducted by experts in the field to identify platform types and select platforms with the largest crowds for further empirical analysis. The platform types were microtask (typical tasks include image captioning, surveys, categorizing data, or simple texting), content creation (typical tasks include creation of extensive texts or translations), micro-sensing (typical tasks include photographing product placements, verification of geo-data or opening hours, or test purchases), and programming (typical tasks include testing and development of software or websites; for platform differentiation, see Leimeister et al., 2016). A questionnaire was administered to and answered by *N*=803 crowdworkers (~200 per platform) asking about their experiences with CW as well as somatic symptoms. The questionnaire was part of a larger interdisciplinary research project answering various research questions concerning CW in Germany (for panel description, Giard et al., 2021). 55 participants had to be excluded from further analysis due to missing responses to some of the demographic variables concerning their work on CW platforms. This resulted in a final sample of *N*=748 crowdworkers (*n*=187 in microtask, *n*=193 in content creation, *n*=186 in micro-sensing, and *n*=182 in programming).

Of the total sample, 56.9% were male, with a medium age of 36.75 (SD=12.03). The average number of hours participating in CW per week was 8.31 (SD=10.31) and medium experience was 35.89months (SD=33.24). Total working hours had a medium of 32.3 (SD=20.2; Median=39.0) hours per week. Table 1 gives an overview of sample demographics.

**Table 1:** Sociodemographic characteristics of participants.

Characteristic	Microtask		Content creation		Microsensing		Programming	
	*n*	%	*n*	%	*n*	%	*n*	%
**Gender**								
Female	94	50.3	118	61.1	55	29.6	56	30.8
Male	93	49.7	75	38.9	131	70.4	126	69.2
Age^a^	36.91 (11.85)	–	43.58 (11.49)	–	34.09 (8.84)	–	31.84 (11.99)	–
**Employment status^b^**								
Employed	92	49.2	38	19.7	128	68.8	93	51.1
Marg. employed	16	8.6	16	8.3	24	12.9	10	5.5
Self-employed	37	19.8	128	66.3	19	10.2	32	17.6
Job training	34	18.2	21	10.9	37	19.9	65	35.7
Unemployed	7	3.7	6	3.1	6	3.2	8	4.4
Not working	8	4.3	8	4.1	4	2.2	0	0
Family-care	16	8.6	17	8.8	5	2.7	6	3.3
Retired	5	2.7	11	5.7	4	2.2	4	2.2
**Highest education**								
University degree	90	48.1	111	38.9	64	34.4	78	42.8
Vocational training	42	22.5	58	30.1	72	38.7	40	22.0
High school	31	16.6	13	6.7	21	11.3	17	9.3
None	24	12.8	11	5.7	29	15.6	47	25.8
**CW**								
Main job^c^	26	13.9	83	43.0	12	6.5	16	8.8
Hours per week^a^	6.8 (7.16)	–	18.85 (12.45)	–	3.83 (4.63)	–	3.3 (5.76)	–
Total work hours^a^	30.40 (21.66)	–	36.85 (18.92)	–	33.79 (18.08)	–	27.91 (21.00)	–
**Platform activity**								
On only one	50	26.7	48	24.9	21	11.3	70	38.5
On 2–3	114	61.0	119	61.7	87	46.7	91	50.0
On 4 or more	23	12.2	26	13.5	78	41.9	21	11.3
**Tasks per week**								
1–10	104	55.6	117	60.6	148	79.6	169	93.9
11–50	67	35.8	71	36.8	34	18.2	8	4.4
>50	16	8.6	5	2.6	4	2.2	3	1.7
Months of partici-pation in CW^a^	24.47 (26.89)	–	63.93 (38.85)	–	29.58 (24.2)	–	24.32 (21.62)	–

*N=748 (n=187 in microtask, n=193 in content creation, n=186 in microsensing, and n=182 in programming). Marg. Employed=marginally employed; CW=crowdwork*.

a *Represents the average value (SD)*.

b *Multiple answers possible*.

c *Represents number of participants answering “yes.”*

### Measures

All items were self-report and presented in German. Participants were informed about data rights and protection and asked about their consent for data storage and analysis. The questionnaire was approved by the ethics counsel of the university prior to administration (identification number #2018-178).

*Experience of CW* was measured with items developed by the researchers. Total work hours per week was measured with one item (“How many hours per week are your working hours including overtime?”), participation in CW was measured with one item asking about the hours doing CW (“How many hours per week on average do you work on crowdworking-platforms?”). To calculate the share, the relation of work hours in CW of total work hours was calculated (hours CW / total work hours). The primary motivation to earn income through CW was asked with one item rating on a 5-point Likert scale ranging from (1) does not apply at all to (5) completely agree (“Why do you work on crowdworking platforms? – Because it is an important source of income”).

*Somatic health* was measured with the Somatic Symptoms Scale (SSS-8; Gierk et al., 2014). The SSS-8 comprises of 8 Items measuring different aspects of somatic health, for example “During the past 7days, how much have you been bothered by any of the following problems: Headache,” measured on a 5-point Likert scale ranging from (1) not at all to (5) very strong (Cronbach’s alpha=0.81). In order to compare the somatic health scores of the CW sample (measured on a Likert scale of 1–5) with the norm sample (Gierk et al., 2014; measured on a Likert scale of 0–4), it was necessary to adjust the scale to the norm sample. A total score of the SSS-8 values was calculated by adding up the item values; therefore, the scale values can range from min=0 to max=32, with higher values indicating more somatic symptoms.

*Work-Life conflict* was measured with the German version (Abendroth et al., 2014) of the Work–Family Conflict Scale (Carlson et al., 2000). Two dimensions of work-life conflict were measured on a 5-point Likert scale ranging from (1) does not apply at all to (5) completely agree, with three items each: time-based work-life conflict (e.g., “I miss important leisure activities with my partner, family, and friends due to my workload”; Cronbach’s alpha=0.89), and strain-based work–family conflict (e.g., “When I come home after work, I often lack the energy for private activities and commitments.,” Cronbach’s alpha=0.84).

### Data Analysis

We applied a two-fold data analysis approach to test our research hypotheses. First, to test hypothesis 1, we applied one-sample *t*-tests to test the differences in somatic symptoms between a norm sample (Gierk et al., 2014) and our crowdworkers sample. Gierk et al. (2014) recruited a representative sample of the German population (*N*=2,510) for their survey validation study. The demographic data of the norm sample shows an even gender distribution with an average age of 49years (higher than the CW sample; M_subsamples_=32–44years); the CW sample shows a slightly higher educational level. Furthermore, we applied hierarchical regression analysis to determine whether the increase of somatic symptoms is attributable to participation in CW and its consequences on work-life conflict. Second, to test our model of crowdworkers’ general somatic health (hypotheses 2–5), we applied moderated mediation analysis (model 8) following recommendations by Hayes (2018). As indicated in hypotheses 4 and 5, we conducted separate analyses to test the effects of the two moderators. Analysis was conducted in SPSS 27.

We report values for the general CW sample (*N*=748) as well as for the subsamples per CW platform type. This approach is reasonable because although the characteristics of the subsamples differ in terms of task type, gender, and age distribution (among others, see Table 1), all crowdworkers experience the same effects when confronted with the characteristics of CW. These characteristics include working remotely and anonymously without direct contact with other crowdworkers or crowdsourcers, deciding autonomously how much and when to work, and being paid only after positive approval. Furthermore, we calculated the values for kurtosis and skewness, since the scales tend to have a non-normal distribution (kurtosis: hours CW=4.19, total work hours=−0.68, share=−1.56, motivation=−1.18, WLC-time=−0.66, WLC-strain=−0.42, somatic symptoms=1.91, SE=0.18; skewness: hours CW=2.01, total work hours=−0.06, share=0.49, motivation=0.00, WLC-time=0.34, WLC-strain=0.48, somatic symptoms=1.27, SE=0.09). However, as the selected methods were robust to deviations from normality, we were able to proceed with the analysis.

## Results

### Descriptive Results

Means, standard deviations, and correlations of study variables are portrayed in Table 2. As expected, a higher amount in hours of participation in CW (*r*=0.09, *p*<0.05), and a higher share of CW in total work hours (*r*=0.13, *p*<0.01) are significantly related with increased somatic symptoms. Also as expected, work-life conflict is significantly positively related with somatic symptoms (*r*_WLCtime_=0.21, *p*<0.01; *r*_WLCstrain_=0.32, *p*<0.01). Interestingly, total work hours is negatively related to somatic health (*r*=−0.08, *p*<0.05). Furthermore, hours of participation in CW and share of CW in total work hours are negatively related with work-life conflict (*r*=−0.02 to −0.21), but positively with somatic symptoms (*r*=0.09 to 0.13), leading to the conclusion that the high flexibility of CW indeed helps crowdworkers to better integrate work on the one side, and family and leisure activities on the other, but is still significantly related to an increase of somatic symptoms.

**Table 2:** Correlations of study variables.

Variable	*M*	*SD*	1	2	3	4	5	6	7	8
1. Sex^a^	56.9	--								
2. Age	36.70	11.95	−0.18^**^							
3. Hours CW	8.32	10.31	−0.18^**^	0.32^**^						
4. Total work hours	32.30	20.20	0.07^*^	0.24^**^	0.29^**^					
5. Share	0.43	0.41	−0.13^**^	−0.02	0.38^**^	−0.67^**^				
6. Motivation	3.02	1.35	−0.23^**^	0.11^**^	0.49^**^	0.00	0.31^**^			
7. WLC-time	2.52	1.09	−0.01	−0.10^**^	−0.02	0.24^**^	−0.21^**^	0.03		
8. WLC-strain	2.34	1.00	−0.02	−0.13^**^	−0.09^*^	0.12^**^	−0.14^**^	0.05	0.70^*^	
9. Somatic health	6.07	5.19	−0.19^**^	−0.05	0.09^*^	−0.08^*^	0.13^**^	0.15^**^	0.21^**^	0.32^**^

*N=748; CW=crowdwork; Share=share of CW in total work hours; Motivation=primary motivation to earn money through CW; WLC-time/−strain=time-based and strain-based work-life conflict; Somatic health=total score on SSS-8 scale*.

a *Represents percentage of males*.

* *p<0.05;*

** *p<0.01*.

### General Somatic Health of Crowdworkers

To answer the first hypothesis whether crowdworkers show more somatic symptoms than a comparison group of regular employed employees, we conducted one-sample *t*-tests (see Table 3), testing the reported means of the norm sample, which were differentiated for males and females (Gierk et al., 2014) against the distributions of the crowdworkers sample. For the male sample, Gierk et al. (2014) reported a mean of 2.94, while male crowdworkers had a mean of 5.20, leading to *t*(425)=9.57, *p*<0.001, *d*=0.46. For the female sample, Gierk et al. (2014) reported a slightly higher mean of 3.29, while female crowdworkers also had a higher mean of 7.21, leading to *t*(323)=13.09, *p*<0.001, *d*=0.73. The result was stable for the analysis per crowdwork platform. We further exploratory tested whether there is an effect of age, even if we did not have a specific hypothesis on the relationship. Gierk et al. (2014) reported an overall correlation of *r*=0.32 (95% CI=0.28–0.35), indicating an increase of symptoms with age. In contrast, the overall correlation of age and somatic symptoms of the total crowdworkers sample was negative and not significant (*r*=−0.05, *p*=0.183), as was the tendency per platform type and gender group.

**Table 3:** Results of mean differences analyses of SSS-8 mean sum scores of the crowdworker sample compared to the norm sample per crowdwork platform & relation with age.

Platform	*n*	Norm Sample	Crowdworker sample		*t*	*p*	Cohen’s *d*	*r* age x SSS-8
		*M*	*M*	*SD*				
Total male	425	2.94	5.20	4.88	9.57	0.001	0.46	−0.00
Total female	323	3.29	7.21	5.38	13.09	0.001	0.73		−0.19^**^
Content creation male	75	2.94	5.85	4.54	5.56	0.001	0.64	0.01
Content creation female	118	3.29	6.80	5.11	7.46	0.001	0.69		−0.20^*^
Microtask male	93	2.94	5.00	4.76	4.17	0.001	0.43	−0.10
Microtask female	94	3.29	7.48	5.71	7.11	0.001	0.73		−0.21^*^
Micro-sensing male	131	2.94	5.02	4.89	4.86	0.001	0.43	0.13
Micro-sensing female	55	3.29	7.13	5.64	5.04	0.001	0.68		−0.06
Programming male	126	2.94	5.16	5.16	4.83	0.001	0.43	−0.11
Programming female	56	3.29	7.70	5.17	6.38	0.001	0.85		−0.19

*1-Sample t-test analysis*.

* *p<0.05;*

** *p<0.01*.

We applied hierarchical regression analysis to determine whether the increase of somatic symptoms in crowdworkers was indeed due to their participation in crowdwork. In three steps, we entered a) age and gender, b) number of hours conducting CW and total work hours per week, and c) time- and strain-based work-life conflict into the hierarchical regression (see Table 4). In total, the chosen indicators explain 16.3 percent of the variability in somatic symptoms. Hours of CW and consequent work-life conflict explained a small, but significant amount of variability in somatic symptoms (ΔR^2^=0.01, *p*<0.05 and ΔR^2^=0.11, *p*<0.001), yet gender and age, otherwise typical determinants of health, also explain only small amounts of variability (ΔR^2^=0.04, *p*<0.001). Therefore, hypothesis 1 can be confirmed, crowdworkers show more somatic symptoms than the norm sample, and an increase of somatic symptoms is due to participation in CW.

**Table 4:** Hierarchical regression results for somatic symptoms.

Variable	*B*	95% CI for B		SE B	*β*	*R* ^2^	ΔR^2^
		LL	UL				
Step 1						0.044	0.04^***^
Constant	10.83	9.02	12.64	0.92			
Gender	−2.17	−2.91	−1.42	0.38	−0.21^***^		
Age	−0.04	−0.07	−0.01	0.02	−0.09^*^		
Step 2						0.056	0.01^*^
Constant	10.82	9.01	12.63	0.92			
Gender	−1.93	−2.69	−1.16	0.39	−0.18^***^		
Age	−0.04	−0.08	−0.01	0.02	−0.10^*^		
Hours CW	0.06	0.02	0.09	0.02	0.11^**^		
Total work hours	−0.02	−0.04	0.00	0.01	−0.07		
Step 3						0.163	0.11^***^
Constant	5.85	3.84	7.85	1.02			
Gender	−1.65	−2.37	−0.93	0.37	−0.16^***^		
Age	−0.02	−0.05	−0.01	0.02	−0.05		
Hours CW	0.07	0.04	0.11	0.02	0.15^***^		
Total work hours	−0.04	−0.05	−0.02	0.01	−0.14^***^		
WFC-time	−0.00	−0.46	0.45	0.23	0.00		
WFC-strain	1.75	1.27	2.24	0.25	0.34^***^		

*CI, confidence interval; LL, lower limit; UL, upper limit; Hours CW, hours doing crowdwork per week; WLC-time/−strain, time-based and strain-based work-life conflict*.

* *p<0.05;*

** *p<0.01;*

*** *p<0.001*.

### Model of Crowdworkers’ General Somatic Health

To test hypotheses 2–5, we conducted moderated mediation modeling in two separate analyses, one for each moderator (see Tables 5–7). Against our prediction in hypothesis 2, somatic health symptoms were significantly negatively related to total work hours per week (*b*=−0.04, *p*=0.040, respectively, *b*=−0.10, *p*=0.000). For the mediation hypotheses in hypothesis 3, we found a relation of total work hours with each time-based (0.02, *p*<0.001) and strain-based work-life conflict (0.01, *p*=0.005, respectively, *p*=0.009), but only strain-based work-life conflict had a significant relation with somatic symptoms (strain: 1.80, respectively, 1.66, *p*<0.001; time: 0.04, *p*=0.877, respectively, 0.07, *p*=0.752). The further moderation analysis of hypothesis 4 (Table 5) showed that the share of CW in total work hours had a significant negative moderating relation with strain-based work-life conflict (strain: −0.02, *p*=0.005), indicating that against our assumptions a higher share of CW indeed offers flexibility. The conditional effects (Table 7) show that for lower shares of CW in total work hours, total work hours rather lead to strain-based work-life conflict, and consequently to somatic health symptoms, whereas the effect of higher shares of CW was inconclusive. Furthermore, we found a positive moderating relation of CW share with somatic health (0.08, *p*=0.009), indicating that higher shares of CW are associated with higher somatic symptoms. The conditional effects (Table 7) also show that a lower share of CW in total work hours is associated with lower somatic health symptoms, whereas a higher proportion reverses the relationship and a higher share is associated with more severe somatic health symptoms. In hypothesis 5 (Table 6), we tested for a moderating effect of the motivation to earn money through CW. Results showed a significant positive moderation on the direct effect only (0.02, *p*=0.0027). The conditional effects show that the negative relationship between total work hours and somatic health symptoms is larger, when the primary motivation to conduct CW is not to earn money. When crowdworkers participate in CW to earn money, the number of total work hours does not explain somatic symptoms. Thus, hypothesis 2 cannot be supported, hypothesis 3 was partially supported for strain-based work-life conflict, and hypotheses 4 and 5 were also each partially supported.

**Table 5:** Moderated mediation analyses for the effect of total work hours on somatic health, mediated by work-life conflict, and moderated by share of CW in total work hours.

	Criterion								
	M_1_ WLC-time			M_2_ WLC-strain			Y Somatic Health		
Antecedent	*B*	*SE*	*p*	*B*	*SE*	*p*	*B*	*SE*	*p*
X total work hours	0.02	0.00	0.000	0.01	0.00	0.005	−0.04	0.02	0.040
M_1_ WLC-time	—	—	—	—	—	—	0.04	0.23	0.877
M_2_ WLC-strain	—	—	—	—	—	—	1.80	0.25	0.000
W_1_ Share	0.08	0.22	0.710	0.20	0.20	0.329	0.19	0.99	0.847
X x W_1_	−0.01	0.01	0.068	−0.02	0.01	0.005	0.08	0.03	0.009
Constant	2.08	0.19	0.000	2.05	0.17	0.000	2.31	0.93	0.013
	*R*^2^ =0.07			*R*^2^ =0.03			*R*^2^ =0.15		
	*F*(3)=17.39, *p* <0.001			F(3)=7.87, *p* <0.001			*F*(5)=24.22, *p* <0.001		

*WLC-time/−strain=time-based and strain-based work-life conflict; Share=Share of crowdwork in total work hours; 95% CI=95% Confidence Interval*.

**Table 6:** Moderated mediation analyses for the effect of total work hours on somatic health, mediated by work-life conflict, and moderated by motivation to earn money through CW.

	Criterion								
	M_1_ WLC-time			M_2_ WLC-strain			Y Somatic Health		
Antecedent	*B*	*SE*	*p*	*B*	*SE*	*p*	*B*	*SE*	*p*
X total work hours	0.02	0.00	0.000	0.01	0.01	0.009	−0.10	0.02	0.000
M_1_ WLC-time	—	—	—	—	—	—	0.07	0.23	0.752
M_2_ WLC-strain	—	—	—	—	—	—	1.66	0.25	0.000
W_2_ Motivation	0.15	0.06	0.011	0.10	0.05	0.053	−0.20	0.26	0.452
X x W_2_	−0.00	0.00	0.017	−0.00	0.00	0.138	0.02	0.01	0.002
Constant	1.66	0.19	0.000	1.84	0.18	0.000	3.59	0.92	0.000
	*R*^2^ =0.06			*R*^2^ =0.02			*R*^2^ =0.14		
	F(3)=17.30, *p* <0.001			F(3)=4.76, *p* =0.003			F(5)=24.82, *p* <0.001		

*WLC-time/−strain=time-based and strain-based work-life conflict; Motivation=primary motivation to earn money through CW; 95% CI=95% Confidence Interval*.

**Table 7:** Conditional direct and indirect effects of the moderated mediation analyses for W_1_ share and W_2_ motivation.

Effect	W_1_ Share	DV: Somatic Health		W_2_ Moti-vation	DV: Somatic Health	
		*b (SE)*	95% CI		*b (SE)*	95% CI
Direct: Total → DV	0.039	−0.04 (0.02)	[−0.07; −0.00]	1.0	−0.07 (0.02)	[−0.11; −0.04]
	0.225	−0.02 (0.02)	[−0.05; 0.01]	3.0	−0.03 (0.01)	[−0.05; −0.01]
	1.000	0.04 (0.02)	[0.00; 0.08]	5.0	0.01 (0.02)	[−0.02; 0.04]
Indirect: Total → WLC-time → DV	0.039	0.00 (0.00)	[−0.01; 0.01]	1.0	0.00 (0.01)	[−0.01; 0.01]
	0.225	0.00 (0.00)	[−0.01; 0.01]	3.0	0.00 (0.00)	[−0.01; 0.01]
	1.000	0.00 (0.00)	[−0.01; 0.00]	5.0	0.00 (0.01)	[−0.00; 0.00]
Indirect: Total → WLC-strain → DV	0.039	0.02 (0.01)	[0.01; 0.04]	1.0	0.02 (0.01)	[0.00; 0.03]
	0.225	0.01 (0.01)	[0.00; 0.03]	3.0	0.01 (0.00)	[0.00; 0.02]
	1.000	−0.01 (0.01)	[−0.03; 0.00]	5.0	0.00 (0.01)	[−0.01; 0.02]

*W_1,2_ =Moderator per Analysis H4 and H5; DV=dependent variable; Total=Total work hours; WLC-time/−strain=time-based and strain-based work-life conflict, Share=Share of crowdwork in total work hours; Motivation=primary motivation to earn money through CW; 95% CI=95% Confidence Interval*.

## Discussion

In the present research, we investigated whether participation in CW is associated with impaired health and by which mechanisms this relation occurs. Results of a sample of *N*=748 crowdworkers across the full scope of German platform types show, that even though CW offers its participants many positive opportunities (e.g., high flexibility of work place, time, and amount of work, easy access for disabled people), participation in CW comes at the price of increased somatic symptoms. Crowdworkers show more somatic symptoms than the norm sample of the questionnaire (Gierk et al., 2014), although their average age was lower. This result is stable across platforms, gender, and age groups, and the effect is directly due to participation in CW. The explained variance is small, but that is often observed when explaining health (e.g., Sparks et al., 1997). For the crowdworker sample, we found no correlation between age and somatic symptoms, suggesting that somatic symptoms do not worsen with age, but that younger crowdworkers in particular exhibit more severe symptoms than their counterpart in the norm sample. Furthermore, we found evidence for our reasoning based on COR-principles (Hobfoll et al., 2018) that negative health effects of CW are due to a lack of regeneration, because CW is usually performed alongside other employment. Many crowdworkers conduct CW as a side-job in their free-time to a main employment (Pesole et al., 2018; Serfling, 2019). Accordingly, total work hours were related to impaired health *via* lack of regeneration represented in strain-based work-life conflict.

CW is therefore relatable to other forms of atypical employment (e.g., Quinlan et al., 2001; Sverke et al., 2002; Tompa et al., 2007; Tavares, 2017) not just in characteristics (e.g., high uncertainty due to short contracts, social isolation in home office, musculoskeletal problems due to inadequate working conditions) but also in its effects on somatic health. Results fit the conclusion of Lewchuk et al. (2008) that employment relations that are characterized by high employment uncertainty and high effort for future employment (such as CW) show the highest health risks. Findings also relate to those of involuntary self-employment (Binder and Coad, 2016). When more socially vulnerable individuals conduct CW in the first place, because its high flexibility fits their requirements for earning money, they must also bear its challenges.

In contrast to our assumptions, total work hours was negatively related to increased somatic symptoms. Crowdworkers with higher total work hours might be more financially stable, and therefore can more freely decide when to take on tasks and when to take regeneration time, and do not have to worry about their financial situation. In contrast, crowdworkers with less total work hours have accordingly a lower income and thus might feel pressured to take on many tasks and tend to overwork to improve their income. Also, underemployment might explain why crowdworkers who experience impaired health do not withdraw from CW. In addition, high total work hours only proved problematic when leading to strain in crowdworkers as demonstrated in the lack of regeneration. High total work hours are related to strain-based work-life conflict, which in turn is related to somatic symptoms, likely because the high workload is energy draining for the crowdworkers. High total work hours are also related to time-based work-life conflict, but time-based work-life conflict is not related to somatic symptoms. Thus, while crowdworkers find it difficult to set aside time for meetings with friends and family or for hobbies, these time constraints are not associated with health constraints, likely because crowdworkers choose this form of employment and therefore experience some form of autonomy in shaping their work.

As the motivation to earn money from CW increases, the negative relationship between total work hours and somatic health weakens. High total work hours is related to somatic symptoms *via* strain-based work-life conflict, but only for crowdworkers who indicated that making money was not their primary motivation for participating in CW. For crowdworkers with a strong motivation to earn money from CW, the direct and indirect effect was not significant. One explanation for this relationship is that individuals who are not as dependent on the money from CW evaluate their activity based on stricter criteria (e.g., effective work-life balance, self-fulfillment from the activity) than individuals who are more dependent on the income from CW. In this line, Brawley (2017) found that with increased seriousness of participating in CW (earning money instead of having a fun activity), the effects of psychological need satisfaction become less accentuated, because external motivators such as earning income are more important. Moreover, the relationship between total work hours and somatic health changes from negative to positive as the share of CW in total work hours increases, suggesting that high total work hours lead to somatic symptoms when they consist only of hours spent in CW. For crowdworkers whose share of crowdwork is low and who are predominantly engaged in another employment, the relationship is mainly determined by that other main job, and higher total work hours are related to a decrease in somatic symptoms. In contrast, if the share is high and crowdworkers work most or all of their work hours as crowdworkers, then high total work hours are related to an increase in somatic symptoms. This effect once again underscores that crowdworkers are a diverse population. Crowdworkers who work (almost) exclusively in CW might mainly belong to vulnerable subgroups, such as individuals with disabilities, who advertise their self-employment through CW, or who are in a transitional phase (e.g., unemployment, between jobs, in vocational training). Among all crowdworkers, those with the highest share are confronted the most with the downsides of CW (e.g., high anonymity, lack of long-term planning opportunities) that cannot be compensated by, for example, the social network or a long-term employment contract of some other employment. Moreover, crowdworkers with high shares may have to work more hours than would be healthy to earn a decent income.

### Future Research Directions

Our results that crowdworkers show increased somatic symptoms offer many interesting research directions, especially concerning the more in-depth analyses of the diversity of the CW population, mechanisms of the relation, and long-term effects.

The CW sample is diverse, therefore crowdworkers differ in their life circumstances (e.g., being temporarily unemployed, being unable to work other jobs due to disability) and motivation to participate in CW (e.g., looking for a fun activity, finding another channel to diversify work of an already self-employed person). Therefore, future research should investigate which subsamples can be distinguished and how CW is related to health impairment in each of these groups. This approach would increase our understanding of the relation of CW and health and allow for sufficient actions.

For the systematic analysis of the relation of CW and health, an extension of the approach chosen in this study of analyzing the role of time shares of total work hours is to analyze psychological characteristics of CW tasks that develop a health enhancing or health decreasing effect. Promising pathways to increase well-being of crowdworkers is the design of CW tasks following work design characteristics to develop meaningful and diverse tasks (Schulte et al., 2020). Further research should also be directed at the role of uncertainty that comes with underemployment and that should be resolved with CW. The Covid-19 pandemic brought about many changes in how and where work is done, and many of these changes will remain in the future (e.g., work from home or mobile work). Research on work from home during the pandemic indicates comparable effects on stress and work–family conflict as we found in our CW sample (e.g., Converso et al., 2021; Galanti et al., 2021). Therefore, there may be parallels in mechanisms between our CW sample and workers who had to work from home during the pandemic and faced jobs that were sparsely regulated in terms of ergonomics and work hours. In addition, individuals may have turned to CW during the pandemic due to short-time work or job losses for whom this type of work would not have been attractive previously. Future research should examine each scenario and explore the parallels between mobile work and CW in terms of the health and demographic characteristics of newly recruited crowdworkers. Long-term effects of the participation in CW must be understood. Our results represent a snapshot of relations of crowdworkers who on average have participated in CW for a couple of years. Future research should use longitudinal analyses to investigate how health impairment effects develop over time (e.g., do the effects worsen, or on the contrary, do crowdworkers adjust and use CW as a positive resource to craft their work experience? e.g., de Bloom et al., 2020), especially in relation to their current life circumstances.

### Practical Implications

The recognition of health impairment effects of CW leads to the question of which support measures need to be established to protect crowdworkers from adverse effects. Two kinds of support are possible: employment regulations and trainings for crowdworkers.

Employment regulations have been shown to be effective in reducing health risks in other domains than CW (Park and Baek, 2019). Regulations for CW stand in the tension between keeping the level of flexibility CW offers and crowdworkers want, but at the same time protecting crowdworkers from risks on the one side, and economic considerations on the other side that require CW to provide quick, easy, and cheap task completion. Therefore, if the CW market in industrialized nations, that most likely will be the first to introduce labor regulations, becomes too limited, on a digital job market tasks will instead be offered globally in less restricted countries, increasing health impairment problems for crowdworkers elsewhere. But which measures can be taken? The securement of a regular labor income was associated with lower health impairment (Lim et al., 2015). The establishment of fair compensations for tasks might relieve crowdworkers from overwork to secure a reasonable income and in effect lead to lower health impairment. Also, CW specific challenges need to be addressed, such as that crowdworkers’ completed tasks can be rejected by crowdsourcers and not get paid at all. Here, better measures of social security become necessary (for more information, see Berg, 2015).

Crowdworkers can also be skilled in designing their own work schedule in a health-promoting manner. In online trainings that could be administered *via* the platforms, they could be informed of the risks of overwork, and supported in detecting their best times for regeneration. In this way, crowdworkers are empowered to craft their own CW work design (e.g., de Bloom et al., 2020). Because platform providers seek ways to commit crowdworkers to their platforms, and experiencing health impairments could lead crowdworkers to terminate their participation in total or at least on a given platform, it would be in the platform providers’ own interests to help crowdworkers reduce these risks.

### Strength and Limitations

To our knowledge, the present study is the first to systematically analyze the impact of participation in CW on crowdworkers’ somatic health across different platform types. Other studies have turned to CW samples for their research, but the intention was to draw conclusions on health effects from a general sample (Créquit et al., 2018). From our study, we must conclude that, in terms of somatic health, crowdworkers may not represent the population as well as demographic analysis revealed (Behrend et al., 2011).

The major limitation of our study is that due to ethical considerations it was not possible to ask about the general health status of crowdworkers prior to their participation in CW. CW offers good opportunities for already health impaired individuals, as amount of work as well as time and place of work can be decided upon at a daily level and in accordance with momentary needs (Zyskowski et al., 2015; Hara and Bigham, 2017). Yet, with our results we cannot identify the percentage of our sample for whom this reasoning may apply. An argument that the percentage might not be very high is that our results are stable across platforms, gender, and age groups. We suggest for future research to replicate our results on healthy and health-impaired samples to validate our results.

Our questionnaire was administered by self-report to a German sample. This approach was chosen because it is less intrusive for participants compared to physical examinations and they can remain anonymous. Also, CW represents digitally enabled work, therefore its participants are best contactable *via* the internet on CW platforms. Self-report measures are generally prone to bias when study participants under- or overestimate their perception (Podsakoff and Organ, 1986). In the case of our CW sample, it is possible that participants, when asked about their life circumstances, wanted to point out the structural problems of CW. Still, other researchers (Shapiro et al., 2013) found crowdworkers to be a reliable source to study psychopathologies and also the norm sample against which our results were compared (Gierk et al., 2014) were administered by self-report. However, for the argument of lack of regeneration, we did not measure regeneration directly, but rather work-life conflict, which seems to us to be an appropriate concretization of the construct to the specific characteristics of crowdworkers. Future research, however, should further investigate the specific relationships between regeneration and its sub-dimensions and health among crowdworkers. In addition, our scales tended not to be normally distributed. Lastly, our results were obtained from a German crowdworker sample; therefore, the results account for crowdworkers from industrialized nations only.

## Conclusion

CW is a new form of digitally enabled work where participants complete tasks online on CW platforms, ranging from tasks of mere seconds to days. For crowdworkers, CW offers a high level of flexibility as they can work when, where, and how much they want. Our results show the downside of this flexibility: Crowdworkers show increased somatic symptoms that are directly related to their participation in CW. Therefore, CW shares not only a characteristic relatedness to other forms of atypical work, but also empirical results prove related risks. Labor regulations that focus on fair payment and training can help in reducing the health impairment risks of crowdworkers.

## Data Availability Statement

The datasets presented in this study can be found in online repositories. The names of the repository/repositories and accession number(s) can be found at: OSF: https://osf.io/ua6xm/?view_only=ec3a94af7ca840509cd1a03eb4a34e4b.

## Ethics Statement

The studies involving human participants were reviewed and approved by Ethics Committee of Bielefeld University. Written informed consent from the participants’ legal guardian/next of kin was not required to participate in this study in accordance with the national legislation and the institutional requirements.

## Author Contributions

KS designed the outline of the study, wrote the draft of the manuscript, and revised it. KS, JS, MR, and GM each contributed ideas to the design of the study. KS, JS, and MR performed statistical analysis. JS, MR, and GM each critically reviewed the drafts. GM supervised this work. All authors contributed to the article and approved the submitted version.

## Funding

This research was funded by the Ministry of Culture and Science of the German State of North Rhine-Westphalia within the research programme “Digital Future.” We acknowledge the financial support of the German Research Foundation (DFG) and the Open Access Publication Fund of Bielefeld University for the article processing charge.

## Conflict of Interest

The authors declare that the research was conducted in the absence of any commercial or financial relationships that could be construed as a potential conflict of interest.

## Publisher’s Note

All claims expressed in this article are solely those of the authors and do not necessarily represent those of their affiliated organizations, or those of the publisher, the editors and the reviewers. Any product that may be evaluated in this article, or claim that may be made by its manufacturer, is not guaranteed or endorsed by the publisher.
